# Mixed nontuberculous mycobacteria in an immunocompromised patient with probable progressive multifocal leukoencephalopathy

**DOI:** 10.1016/j.ijregi.2024.100502

**Published:** 2024-11-29

**Authors:** Christoffel Opperman, Janet Scott, Janre Steyn, Sarishna Singh, Yonas Ghebrekristos, Roland Croxford, Robin Warren, Aliasgar Esmail, Giovanni Ghielmetti, Wynand Goosen

**Affiliations:** 1 National Health Laboratory Service, Green Point TB-Laboratory, Cape Town, South Africa; 2 SAMRC Centre for Tuberculosis Research, Division of Molecular Biology and Human Genetics, Faculty of Medicine and Human Genetics, Stellenbosch University, Cape Town, South Africa; 3 Division of Medical Microbiology, Department of Pathology, University of Cape Town, Cape Town, South Africa; 4 DP Marias Hospital, Western Cape Department of Health, Cape Town, South Africa; 5 University of Cape Town Lung Institute, Centre for Lung Infection and Immunity, Division of Pulmonology, Department of Medicine, University of Cape Town and Groote Schuur Hospital, Cape Town, South Africa; 6 Section of Veterinary Bacteriology, Institute for Food Safety and Hygiene, Vetsuisse Faculty, University of Zurich, Zurich, Switzerland; 7 Department of Microbiology and Biochemistry, University of the Free State, Bloemfontein, South Africa

**Keywords:** Nontuberculous mycobacteria, Targeted amplicon-based sequencing, Co-infection, Progressive multifocal leukoencephalopathy

## Abstract

**Objectives:**

Nontuberculous mycobacteria (NTM) are increasingly recognized opportunistic pathogens found ubiquitously in the environment. The presence of multiple NTM species at the site of disease complicates diagnosis and treatment.

**Case and management:**

A 40-year-old patient who tested positive for HIV, with an absolute clusters of differentiation 4+ T-cell count of 3 cells/µl and cryptococcaemia, presented with hemoptysis, productive cough, and weight loss. Mixed NTM species, including *Mycobacterium kansasii* and *Mycobacterium chelonae,* were detected by the GenoType Mycobacterium Common Mycobacteria line probe assay from respiratory specimens, with *Mycobacterium avium* bacteremia also identified in the same month. An empirical regimen of azithromycin, ethambutol, isoniazid, and rifabutin was initiated to address recurrent positive cultures with *M. kansasii* and the NTM bacteremia. Despite this treatment, the patient experienced neurologic deterioration, was diagnosed with probable progressive multifocal leukoencephalopathy, and subsequently died.

**Additional investigation:**

Advanced diagnostic techniques, including Sanger sequencing, Deeplex Myc-TB in combination with short-read next-generation sequencing, and targeted amplicon-based Oxford Nanopore Technologies long-read sequencing, revealed the presence of mixed NTM species in two retrospective stored cultures, with variations in primer binding affinity among the *M. kansasii* and *M. avium*.

**Conclusion:**

This case highlights the individualized considerations required to manage a patient with mixed NTM infection and the need for multi-target diagnostic approaches.

## Highlights

• Concurrent pulmonary and disseminated nontuberculous mycobacteria (NTM) disease can involve multiple species.
• Line probe assays may fail to detect NTM mixtures from pulmonary specimen cultures.
• Mixed NTM infections can be either pathogenic or non-pathogenic.
• Multi-target amplicon sequencing is better for detecting NTM mixtures.
• Dual NTM treatment is complex, requiring a personalized regimen and expert input.

## Introduction

Nontuberculous mycobacteria (NTM) represent an overlooked yet increasingly recognized opportunistic disease that is gaining global attention [1]. They are ubiquitously found in the environment, with a zoonotic potential [2]. In contrast to *Mycobacterium tuberculosis* complex (MTBC), NTM is rarely transmitted from person to person [3]. Instead, water sources are regarded as common reservoirs for human and animal infection and colonization by NTM [4]. In addition, it has been suggested that livestock can act as a route for NTM transmission to humans through unpasteurized milk and the consumption of meat [2].

NTM mixtures present significant challenges due to their difficult-to-treat nature, varying treatment requirement for different species, lack of clear treatment guidelines, uncertain prognostic outcomes, and limited diagnostic modalities for detecting the presence of NTM mixtures [5,6]. This report highlights the intricate decision-making process involved in diagnosing and managing NTM mixed disease (the presence of more than one clinically relevant NTM species).

## Case and management

A 40-year-old male presented with a chronic productive cough (>2 weeks), non-massive hemoptysis, and weight loss. Chest X-ray revealed hyperinflation and fine reticulonodular infiltrates in the middle and lower zones bilaterally, with no prominent evidence of fibrosis or cavitation. The patient was admitted and subsequently initiated on an empirical rifampicin-based tuberculosis treatment regimen (Rifafour) due to a positive Auramine O stain smear, which revealed a 3+ grade for acid-fast bacilli. He was known to have HIV; however, he had disengaged from care and interrupted antiretroviral treatment. He had clusters of differentiation 4+ T-cell count of 3 cells/µL on admission. Cryptococcaemia was diagnosed with a positive cryptococcal antigen test from serum, although cerebrospinal fluid (CSF) testing was culture and latex agglutination test negative for *Cryptococcus neoformans*, and the patient did not exhibit signs of meningitis. In addition, mycobacteria growth indicator tube (MGIT) (Becton Dickinson, Berkshire, United Kingdom) culture was negative for acid-fast bacilli. Fluconazole 800 mg was started orally and subsequently tapered to 200 mg daily over several weeks.

Mixed NTM were isolated within the same month. Specifically, *Mycobacterium avium* was cultured from a blood sample, whereas *Mycobacterium kansasii* and *Mycobacterium chelonae* were identified from separate sputum cultures. GeneXpert MTB/RIF Ultra assay (Cepheid, Sunnyvale, California, USA) was negative for *M. tuberculosis* complex. The commonly used GenoType Mycobacterium Common Mycobacteria (CM) line probe assay (LPA) (Bruker, Billerica, Massachusetts, USA) only detected the *M. kansasii* (cultured on four separate occasions in MGIT cultures) and *M. chelonae* (cultured once in an MGIT) but not *M. avium* (detected with a CM LPA from a blood culture) from the respiratory samples. After consultation with an infectious diseases specialist, management was changed to an oral daily regimen of azithromycin (500 mg), ethambutol (800 mg), isoniazid (200 mg), and rifabutin (450 mg). This treatment was initiated empirically in keeping with international guidelines and based on low local rates of macrolide resistance in South Africa (SA). A month after the initiation of an empiric NTM regimen, the patient was reintroduced to HIV antiretroviral therapy consisting of the oral fixed-drug combination tenofovir (300 mg), emtricitabine (200 mg), and efavirenz (600 mg). While on treatment, he developed a left-sided partial paralysis, ataxic gait, and blurred vision. A computed tomography (CT) scan of the brain showed a right superior cerebellar peduncle infarct. A multidisciplinary team was involved in managing the patient holistically, including a physiotherapist for mobilization, a social worker to assist with a disability grant, and an ophthalmologist for visual impairment due to suspected glaucoma.

However, after initial clinical improvement, additional neurologic symptoms developed after 1 month of the initial CT brain imaging that included dysarthria and upper motor neuron weakness. A repeat CT scan of the brain showed no new lesions. Polymerase chain reaction (PCR) confirmation of John Cunningham virus DNA in the CSF, combined with rapid neurologic deterioration, suggested probable progressive multifocal leukoencephalopathy (PML). The patient and his family were counseled on the disease prognosis, and, shortly thereafter, he died, potentially due to progressive PML. Figure S1 illustrates the chronologic flow of events, including laboratory and imaging investigations, as well as the holistic management of the patient.

## Additional molecular investigations

In addition to the routine GenoType Mycobacterium CM LPA, we performed Sanger sequencing of the *hsp65* gene (heat shock protein of 65 kDa), Deeplex Myc-TB (Genoscreen, Lille, France) targeted next-generation sequencing analysis, and an in house developed [7] targeted amplicon-based next-generation long-read sequencing PCR approach using Oxford Nanopore technology (ONT) (Oxford, UK) on two retrospectively stored respiratory cultured isolates (Figure S1). Details regarding these techniques are provided in the supplementary material, and Table 1 shows the results. The variations in PCR primer binding affinity for *hsp65* are illustrated in Figure S2, explaining the discordant results observed for *M. avium* and *M. kansasii*.

**Table 1:** Comparison of *Mycobacterium* species identification methods on retrospectively stored samples: Sanger sequencing, Deeplex Myc-TB next generation sequencing, in-house targeted amplicon-based next-generation sequencing using Oxford Nanopore Technology, and GenoType Mycobacterium Common Mycobacteria line probe assay.

Diagnostic modality	Respiratory sputum 1 (July 2017)	Respiratory sputum 2 (September 2017)
Sanger sequencing of heat shock protein (*hsp65*) gene		
	*Mycobacterium avium*: 100% gene coverage with 100% identification	*Mycobacterium avium*: 100% gene coverage with 99.4% identification
Deeplex Myc-TB		
	*Mycobacterium kansasii*: 100% identification (sequence depth 62,828)	*Mycobacterium kansasii*: 100% identification (sequence depth 76,262)
Targeted amplicon-based next-generation sequencing using Oxford Nanopore technology		
Heat shock protein *hsp65* gene	*Mycobacterium avium*: 100% proportion percentage identification (reads 105,389)	*Mycobacterium avium*: 100% proportion percentage (reads 110,025)
Ribonucleic acid polymerase beta subunit (*rpoB*) gene	*Mycobacterium kansasii*: 55.8% proportion percentage identification (reads 32,307) *Mycobacterium avium*: 44.2% proportion percentage identification (reads 25,600)	*Mycobacterium kansasii*: 56% proportion percentage identification (reads 26,376) *Mycobacterium avium*: 44% proportion percentage identification (reads 20,720)
GenoType Mycobacterium Common Mycobacteria line probe assay		
	*Mycobacterium kansasii*	*Mycobacterium kansasii*

## Discussion

The change of NTM species and the simultaneous isolation of multiple pulmonary NTM species in the same patient during or after treatment have been well-documented [8]. It has been suggested that at the initial diagnosis of NTM lung disease, these patients already have two or more NTM species in different anatomic regions of the lung [8]. LPAs may potentially overlook these subpopulations within NTM mixtures, as indicated by previous research [6], possibly due to the second NTM falling below the limit of detection. In the current case, an NTM mixture was identified from the initial cultures with next-generation amplicon-based ONT long-read deep sequencing. The presence of *M. avium* in the respiratory sample before its detection in the blood culture suggests that the bacterium may have disseminated from the lung. Our results also indicate that different testing modalities may exhibit varying primer binding affinities for the same target across different species, thereby highlighting the advantage of using a diagnostic test with multiple targets. However, it remains unclear if these initial non-dominant populations and variants should be targeted during the initial stages of treatment to ensure favorable outcomes.

The selection of an appropriate treatment regimen for mixed NTM infections is primarily influenced by four key factors: (i) the clinical status of the patient (critically ill vs non-critically ill), (ii) the site of NTM isolation (sterile vs non-sterile sites), (iii) the specific organism(s) isolated, and (iv) the clinical likelihood that the organism is pathogenic (colonizer vs pathogen). In critically ill patients, particularly, those with advanced immunosuppression, early initiation of treatment is crucial because these patients are at a markedly high risk of mortality. Consequently, clinicians may adopt a lower threshold for initiating empirical treatment that covers all potentially pathogenic NTM organisms, particularly, when the infection is isolated from a sterile site, such as CSF or blood. In many instances, treatment may be initiated empirically based on rapid molecular diagnostics, even in the absence of strict adherence to microbiological diagnostic criteria for NTM pulmonary disease, which typically requires two independent sputum cultures [1]. The treatment regimen for critically ill patients should also take into account potential adverse effects, drug penetration at the site of infection (e.g. CSF), and potential drug interactions, particularly, in patients receiving medications, such as antiretrovirals, that may interact with the cytochrome P450 system.

Although no randomized clinical trials have evaluated treatment regimens for mixed NTM disease [9], combination therapy covering all significant NTM species is considered reasonable in critically ill patients with advanced immunodeficiency. An alternative strategy would be to perform quantitative NTM cultures on solid agar (in centers where this is offered) to assess the burden of disease [10] and treating the dominant NTM species only in cases where the mixed NTM infection requires varying treating. However, in very sick patients, prompt treatment initiation is vital and cultures may introduce unacceptable delays. Furthermore, although drug susceptibility testing for NTM is recommended, it is not always available in resource-constrained settings [5]. In SA, the prevalence of macrolide resistance among NTM in the clinical setting remains low, as demonstrated by a recent study indicating a resistance rate of 2.8% (four of 145) [11]. For this patient, an empiric regimen including both a macrolide and rifamycin was appropriate because it provided therapeutic cover for *M. avium* and *M. kansasii*. These NTM were thought to be the main pathogens in this case and low rates of macrolide resistant *M. avium* isolates is documented in SA [9,11]. Macrolides are essential for treating pulmonary *Mycobacterium avium* complex (MAC), and this agent would also be effective for *M. avium* bacteremia, provided that it is susceptible to azithromycin [9]. *M. kansasii* was managed with a rifamycin-based multidrug regimen, which included rifabutin, as the first-line empiric treatment [9]. Rifabutin is known for its effectiveness against MAC and typically exhibits lower minimum inhibitory concentrations than other rifamycins [12]. In addition, it has a more favorable drug interaction profile, particularly, with antiretroviral drugs used in HIV treatment (less potent inducer of cytochrome P450 enzymes) [13]. Until more evidence becomes available, a multidrug regimen (≥3 drugs) to prevent resistance and cover all NTM species in mixed infections is likely to outweigh the risk of side effects. If *M. kansasii* was repeatedly cultured from sputum after 6 months of guideline-based treatment (indicating a refractory case), drug susceptibility testing should have been prioritized to exclude a rifampicin-resistant strain. Fluoroquinolones could be considered as part of second-line treatment if such a rifampicin-resistant strain was encountered [9]. The role of inhaled (liposomal) or parenteral amikacin combined with guideline-based therapy in mixed NTM disease to improve clinical outcomes, particularly, where MAC is involved in pulmonary non-refractory or extrapulmonary cases, warrants further investigation [14]. The single sputum culture positive for *M. chelonae* was regarded as a contaminant and not targeted during disease treatment because this was only cultured once from an unrelated sputum sample. The non-cavitary lung presentation might have justified a less aggressive thrice-weekly regimen to increase tolerability and potentially decrease the side effects of a multidrug regimen; however, given the patient's severe clinical condition, *M. avium* bacteremia, and advanced immunosuppression, a daily treatment regimen was opted for [9]. In this case report, it seems that the patient did have an initial clinical response to treatment, with a subsequent clinical deterioration attributable to PML. In addition, although there are numerous anecdotal case reports suggesting that mixed NTM disease treatment can be successful in ∼40-60% of patients [15], extrapolating treatment outcomes to critically ill patients with advanced HIV must be interpreted with caution.

Previous studies have shown that commonly used LPAs may fail to detect mixed infections [6], highlighting the importance of obtaining pure cultures for accurate diagnosis. In this context, the use of mycobacteria-selective media, such as the NTM Elite agar (bioMérieux, France) plate, represents a viable alternative. These selective plates not only facilitate the monitoring of disease progression but they also reduce the contamination from surrounding respiratory flora. In addition, they allow the differentiation of colony morphology and pigmentation in mixed NTM infections [16]. After the isolation of single colonies, it is essential to confirm their acid-fastness using a Ziehl-Neelsen stain, after which identification can be carried out using the GenoType Mycobacterium CM/Additional Species LPAs. This approach could be incorporated as a practical recommendation, particularly, in resource-limited settings where next-generation sequencing is not available.

Lastly, a high mortality rate is associated with PML [17]. A retrospective review over a 25-year period identified common symptoms such as hemiparesis (31%), gait instability (25%), and dysarthria (19%), which were also evident in this case [18]. High-resolution clinical imaging of the brain, such as magnetic resonance imaging, was not performed in this case due to resource constraints and could have revealed subtle and more classic changes associated with PML. The cause of death is most likely multifactorial. PML has a poor prognosis. In addition, opportunistic infections resulting from severe immunosuppression secondary to HIV, along with the possibility of immune reconstitution inflammatory syndrome after antiretroviral treatment initiation, were all present. Family members and clinicians declined an autopsy, and, as such, we were unable to directly confirm mixed NTM as the cause of death.

## Conclusion

This case highlights the need for comprehensive diagnostic approaches and the necessity of multidisciplinary teams to formulate personalized treatment strategies to address the complexities of NTM mixed infections. It accentuates the value of a diagnostic platform that encompasses multiple targets and highlights the variability in PCR primer binding affinity across different organisms within the same genus. In addition, it reminds us of the limited data available for treating NTM mixtures, which often relies on anecdotal evidence and is managed on a case-by-case basis with expert consultation.

## Declarations of competing interest

The authors have no competing interest to declare.

## Appendix Supplementary materials

Image, application 1
