# Supplementary material for: Mixed nontuberculous mycobacteria in an immunocompromised patient with probable progressive multifocal leukoencephalopathy
Source: IJID Reg. 2024 Nov 29;14:100502. doi: 10.1016/j.ijregi.2024.100502 (PMC11730941; doi:10.1016/j.ijregi.2024.100502)
Supplement: Supplementary file 1 [file mmc1.docx]

**Mixed nontuberculous mycobacteria in an immunocompromised patient with probable progressive multifocal leukoencephalopathy**

**Supplementary Material**

| **Table of contents** | **Page number** |
| --- | --- |
| 1. DNA extraction for Sanger sequencing, Deeplex®Myc-TB, and Oxford Nanopore Technology, | 1 |
| 2. Sanger sequencing hsp65 gene primers and PCR thermocyler conditions | 2-3 |
| 3. Oxford Nanopore Technology targeted deep amplicon-based sequencing | 3-6 |
| 4. Oxford Nanopore Technologies Bioinformatics | 6-7 |
| 5. Deeplex®Myc-TB analysis | 7-10 |
| Table S1. Primers and thermocycling conditions for targeted amplicon-based sequencing | 11 |
| Figure S1. Timeline of events, encompassing laboratory and imaging investigations, and the overall management approach for the patient | 12 |
| Figure S2. Differences in the genetic composition of *Mycobacterium kansasii* and *Mycobacterium avium* at the binding sites of the *hsp65* forward and reverse primers | 13 |

1. DNA extraction for Sanger sequencing, Deeplex®Myc-TB, and Oxford Nanopore Technology,

Total DNA extraction was performed using a modified protocol based on the DNeasy Blood and Tissue Kit (Qiagen, Hilden, Germany). The process began with the treatment of 1 milliliter (mL) of Mycobacteria Growth Indicator Tube (MGIT) broth (Becton Dickinson, Berkshire, United Kingdom). Initially, the sample was heat-inactivated at 98°C for 45 minutes and then subjected to centrifugation at 1,500 × g for 10 minutes to pellet the cells. Next, 300 microliters (μL) of buffer ATL were added to the cell pellet, and digestion was initiated by incorporating 25 μL of proteinase K. This mixture was incubated overnight at 55°C. After digestion, the sample was centrifuged at 5,500 × g for 5 minutes, and 500 μL of the supernatant was transferred to a 1.5 mL tube. To this supernatant, 400 μL of buffer AL and 400 μL of ethanol were added. The resulting mixture was applied to a Mini Spin column for DNA binding. The isolated DNA was purified with wash buffers AW1 and AW2 and then eluted in 60 μL of AE buffer, prewarmed to 54°C.

2. Sanger sequencing *hsp65* gene primers and PCR thermocyler conditions

The primers for the heat-shock 65 (*hsp65*) gene used were Forward- 5’-ACCAACGATGGTGTGTCCAT-3’ and Reverse- 5’-CTTGTCGAACCGCATACCCT-3’. For each 25 μL PCR reaction, the mixture included 12.5 μL of NEB One Taq® Hot Start 2X Master Mix (New England Biolabs, Massachusetts, USA), 1 μL of each 50 μM primer stock solution, 5.5 μL of sterile nuclease-free water, and 5 μL of undiluted extracted DNA. The thermal cycling protocol commenced with an initial denaturation at 94°C for 10 minutes, followed by 40 cycles consisting of denaturation at 94°C for 30 seconds, annealing at 62.5°C for 30 seconds, elongation at 72°C for 30 seconds, followed by a final elongation at 72°C for 5 minutes, ending with a hold at 4°C for infinity. The target size of the amplified product was confirmed using a 1.5% agarose gel, prepared with 2.25 mg of agarose in 150 ml of 1x Sodium Borate Buffer (SB buffer, pH 8.5) and 7.5 µl of SmartGlow™ dye added. The gel was run in 450 ml of 1x SB buffer, which also contained 1% SmartGlow™ dye, at a voltage of 100 for 45 minutes. Visualization and documentation of the gel were performed with the ChemiDoc M.D. Universal Hood III Gel Documentation System (Bio-Rad Laboratories, Hercules, California, USA).

Amplicons were sent to the Central Analytical Facility at Stellenbosch University, Cape Town, South Africa, for Sanger sequencing. The resulting sequences were pairwise aligned using A Plasmid Editor (ApE; v3.1.3). The consensus sequences were then analyzed using the National Center for Biotechnology Information (NCBI) nucleotide Basic Local Alignment Search Tool (BLASTn). A similarity index and gene coverage of greater than 99% was considered sufficient for accurate identification, and the results were compared with the strain data available in GenBank (http://www.ncbi.nlm.nih.gov/genbank/).

3. Oxford Nanopore Technology targeted deep amplicon-based sequencing

The PCR master mix was prepared by combining 8.5 μL of nuclease-free water, 12.5 μL of NEB One Taq® Hot Start 2X Master Mix (New England Biolabs, Massachusetts, USA), 1 μL of each forward and reverse primer (10 μM, 10 pmol/μL), and 2 μL of extracted DNA, achieving a final reaction volume of 25 μL per sample. Thermocycling conditions and specific primer details are listed in Table S1.

*Native barcoding ligation*

For native barcoding ligation, 12.5 μL of PCR amplicons, quantified at 300 femtomolar (fmol) and verified by gel electrophoresis was mixed with 1.75 μL of NEB Ultra II End Prep Reaction Buffer and 0.75 μL of Ultra II End Prep Enzyme Mix (New England Biolabs, Massachusetts, USA). This mixture was incubated at 20°C for 5 minutes. Subsequently, for the DNA barcoding reaction, a combination of 3 μL of nuclease-free water, 0.75 μL of end-repaired DNA, 1.25 μL of a unique native barcode [sample 1 (Forward- 5’-CACAAAGACACCGACAACTTTCTT-3’, Reverse- 5’-AAGAAAGTTGTCGGTGTCTTTGTG-3’); sample 2 (Forward- 5’-CGTCAACTGACAGTGGTTCGTACT-3’, Reverse- 5’-AGTACGAACCACTGTCAGTTGACG-3’)], and 5 μL of NEB Blunt/TA Ligase Master Mix (New England Biolabs, Massachusetts, USA) was prepared. This mixture was incubated at room temperature for 20 minutes, and the reaction was terminated by adding 1 μL of EDTA.

The barcoded samples, each 11 μL, were pooled into a 1.5 mL low-bind microcentrifuge tube. To purify the pooled reaction, AMPure XP beads (Beckman-Coulter, California, USA) were added at a 0.4X volume ratio and incubated for 10 minutes at room temperature with mixing. Following this, the mixture was placed on a magnetic rack for 5 minutes, after which the supernatant was discarded. The beads were washed with 700 μL of 80% freshly prepared ethanol and briefly dried for 30 seconds. After removing the beads from the magnetic rack, the pellet was resuspended in 35 μL of nuclease-free water and incubated at 37°C for 10 minutes with intermittent agitation. Following this incubation, 35 μL of the clear eluate was transferred to a new 1.5 mL tube.

*Adapter ligation and clean-up*

In the subsequent step, 30 μL of the pooled barcoded culture was combined with 5 μL of Native Adapter, 10 μL of NEB Quick Ligation Reaction Buffer, and 5 μL of Quick T4 DNA Ligase (New England Biolabs, Massachusetts, USA) in a 1.5 mL low-bind microcentrifuge tube. The reaction mixture was incubated at room temperature for 20 minutes. Following the ligation, an AMPure XP cleanup (Beckman-Coulter, California, USA) was performed as previously described. After an additional 10-minute incubation at room temperature with mixing, the culture was subjected to centrifugation on a magnetic rack for 5 minutes, ensuring the eluate was clear. The supernatant was discarded, and the beads were washed with 700 μL of short fragment buffer while on the magnetic rack. The residual buffer was then removed, and the beads were briefly dried for 30 seconds. The dried pellet was resuspended in 7 μL of elution buffer and incubated at 37°C for 10 minutes with intermittent agitation. Following this incubation, 15 μL of the clear eluate was transferred to a new 1.5 mL tube. The cleaned library was quantified using the Qubit Double-Stranded (ds) DNA High Sensitivity Assay Kit (Life Technologies, California, USA) and adjusted to a concentration of 5 fmol in a minimum volume of 12 μL.

*Priming and loading of Flongle*

For the priming and loading of the Flongle flow cell, 1 mL of flow cell priming mix, consisting of 3 μL of flow cell tether and 117 μL of flow cell flush, was introduced into the priming port of the flow cell. Following this, 30 μL of the prepared library, which included 15 μL of sequencing buffer, 10 μL of library beads, and 5 μL of DNA library, was added to the Flongle flow cell R10 Version (83 pores). Sequencing was subsequently carried out using the MinION mk1C device (ONT, Oxford, UK).

4. Oxford Nanopore Technologies Bioinformatics

The analytical process involved the examination of two datasets (samples 1 and 2, as detailed in Table 1) generated from sequencing using the MinION mk1C device (ONT, Oxford, United Kingdom). After data acquisition, base calling (260bps – High-Accuracy), de-multiplexing, and barcode trimming were performed with Guppy [v6.4.6]. The quality of the sequencing reads was evaluated using FastQC (v0.11.9) and pycoQC (v2.5.0.23). Reads with a Q score below 12 were filtered out using Nanoq (v0.10.0). Following quality control, a reference-free read sorting was conducted using the amplicon sorter tool (version 2023-06-19). For each barcode, 200 000 randomly selected reads, ranging in length from 50 to 2,000 base pairs (bp), were analyzed. The consensus sequences were subsequently categorized by amplicon size and genetic similarity, with relative abundances determined from the representative read pool. Sequence screening was performed using the ABRicate software tool, which utilized customized databases for each target to generate summary reports. Sequences demonstrating more than 90% coverage and identity matches greater than 98% were classified to specific mycobacterial species or complexes based on the highest identity match.

5. Deeplex®Myc-TB analysis

The amplicons were generated using the Deeplex®Myc-TB master mix. A volume of 15.8 µl of this master mix was combined with 0.2 µl of a diluted internal control, designed to account for potential PCR inhibition from non-mycobacterial DNA. This mixture was distributed across all wells of the PCR plate, except for the well designated for the negative control, which contained only the master mix. DNA samples from each isolate, with a concentration exceeding 1 pg/µl, were added in 9 µl to the respective wells, while the positive control well received 9 µl of a diluted *Mycobacterium bovis* DNA solution, according to the manufacturers instructions. The PCR reaction was set with a final volume of 25 µl, and the protocol involved preheating the lid to 100°C. The cycling conditions included an initial denaturation at 98°C for 2 minutes, followed by 35 cycles of denaturation at 94°C for 1 minute, annealing at 55°C for 30 seconds, extension at 72°C for 1 minute and 30 seconds, and a final extension at 72°C for 10 minutes, ending with a hold at 4°C.

*Amplicons clean-up*

After PCR amplification, the amplicons underwent a clean-up process. To each well, 75 µl of 10 mM Tris-HCl (pH 7.8) and 65 µl of Agencourt AMPure XP® bead suspension was added. The samples were incubated at room temperature before being placed on a magnetic rack to separate the beads. The supernatant was removed, and the beads were washed twice with freshly prepared 80% ethanol. After removing residual ethanol, the beads were dried for approximately 10 minutes. The beads were then resuspended in 26 µl of 10 mM Tris-HCl (pH 7.8), incubated, and separated magnetically. 25 µl of the purified amplicon DNA was transferred to a new plate, where its quantity and quality were assessed. High-quality DNA was confirmed if the concentration was above 1 ng/µl and the 260/280 ratio was around 1.8.

*Quantification of the purified DNA*

Quantification of the purified DNA was performed using the Qubit double-stranded (ds) DNA High Sensitivity (HS) Assay kit. This assay included both broad-range and high-sensitivity components to ensure accurate quantitation. The negative control was confirmed to be below the detection limit, while the external positive control showed a concentration well above 0.2 ng/µl. The concentration of the cultures was ideally maintained at or above 0.2 ng/µl.

*Tagmentation*

For library preparation, 5 µl of the DNA at a concentration of 20 ng/µl was combined with 20 µl of tagmentation master mix. This mixture was incubated in a thermocycler with the lid preheated to 100°C, at a final volume of 50 µl, with a program that included 55°C for 15 minutes and a hold at 10°C. Post-tagmentation clean-up involved adding 10 µl of tagment stop buffer (TSB) to each well, resuspending the contents, and then performing PCR with preheated lid at 100°C, reaction volume set to 60 µl, and incubation at 37°C for 15 minutes followed by holding at 4°C. The tubes were then placed on a magnetic plate to clear the liquid. The supernatant was discarded, and the beads were washed three times with 100 µl of tagment wash buffer (TWB). For DNA amplification, 40 µl of PCR master mix was added to the beads, and the samples were cycled with specific conditions: reheating the lid to 100°C, setting the reaction volume to 50 µl, and running cycles at 68°C for 3 minutes, 98°C for 3 minutes, followed by 98°C for 45 seconds, 62°C for 30 seconds, 68°C for 2 minutes, 68°C for 1 minute, and holding at 10°C.

*Library clean-up*

The cleaned libraries were then centrifuged at 280 X g for 1 minute, ensuring that the liquid was clear using a magnetic stand. 45 µl of the supernatant was transferred and mixed with 40 µl of nuclease-free water and 45 µl of culture purification beads (SPB). This mixture was incubated at room temperature for 5 minutes, and the tubes were placed on a magnetic bead stand until the liquid was clear. 125 µl of supernatant was transferred to a new plate along with 15 µl of SPB, followed by another 5-minute incubation and magnetic stand usage. The supernatant was discarded, and the beads were washed twice with 200 µl of 80% ethanol. After removing residual ethanol and air-drying for 5 minutes, 32 µl of resuspension buffer was added. The mixture was incubated at room temperature for 2 minutes before transferring 30 µl of supernatant to a new plate.

*Loading MiniSeq system flow cell*

The library was quantified using the dsDNA HS Qubit Assay to achieve a final loading concentration of 1.2 pM. Libraries with a final concentration of 2 nM were adjusted to a final solution of 10 µl. For libraries at or below 2 nM, 10 µl of undiluted library was used. The volumes of individual libraries were pooled into a single tube, with Tris HCl or RSB added to reach the required total volume (number of cultures x 10 µl). The library pool was kept on ice. PhiX was prepared by denaturing with 5 µl of 0.1 N NaOH, neutralizing with 200 mM Tris-HCl, and diluting to a loading concentration with HT1. The final library and PhiX mix was loaded onto the MiniSeq System flow cell, which was thawed for 30 minutes at room temperature before use.

Table S1. Primers and thermocycling conditions for targeted amplicon-based sequencing.

| **Name** | **Forward/**  **Reverse** | **Sequence 5'-3' [1-4]** | **Size (bp)** | **Annealing temperature^a^ (℃)** | **Elongation time^b^** |
| --- | --- | --- | --- | --- | --- |
| *hsp65** | Tb11 (Forward) | ACCAACGATGGTGTGTCCAT | 441 | 62.5 | 30 seconds |
|  | Tb12  (Reverse) | CTTGTCGAACCGCATACCCT |  |  |  |
| *rpoB* † | MycoF (Forward) | GGCAAGGTCACCCCGAAGGG | 764 | 64 | 1 minute |
|  | MycoR  (Reverse) | AGCGGCTGCTGGGTGATCATC |  |  |  |

Denaturation was carried out at 94°C for 15 minutes for 1 cycle, followed by 35 cycles of denaturation at 94°C for 30 seconds, annealing at variable temperatures^a^ for 30 seconds, and elongation at 68°C for variable durations^b^. A final elongation cycle was performed at 72°C for 5 minutes with a hold at 4°C.

* Telenti A, Marchesi F, Balz M, Bally F, Böttger EC, Bodmer T. Rapid identification of mycobacteria to the species level by polymerase chain reaction and restriction enzyme analysis. J Clin Microbiol 1993;31(2):175-8. doi: 10.1128/jcm.31.2.175-178.1993.

† Adékambi T, Colson P, Drancourt M. *rpoB*-based identification of nonpigmented and late-pigmenting rapidly growing mycobacteria. J Clin Microbiol 2003;41(12):5699-708. doi: 10.1128/JCM.41.12.5699-5708.2003.


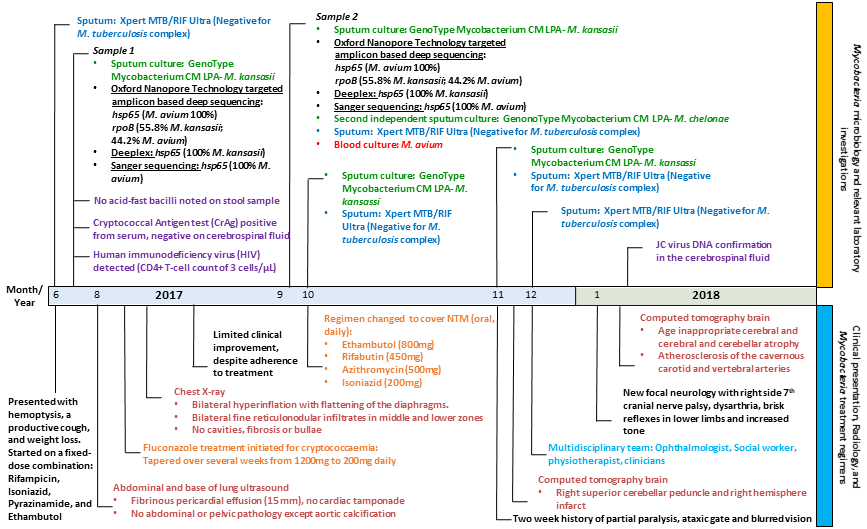


**Figure S1.** Timeline of events, encompassing laboratory and imaging investigations, and the overall management approach for the patient.

CM: Common Mycobacteria; JC Virus: John Cunningham virus; LPA: Line probe assay; NTM: Nontuberculous mycobacteria


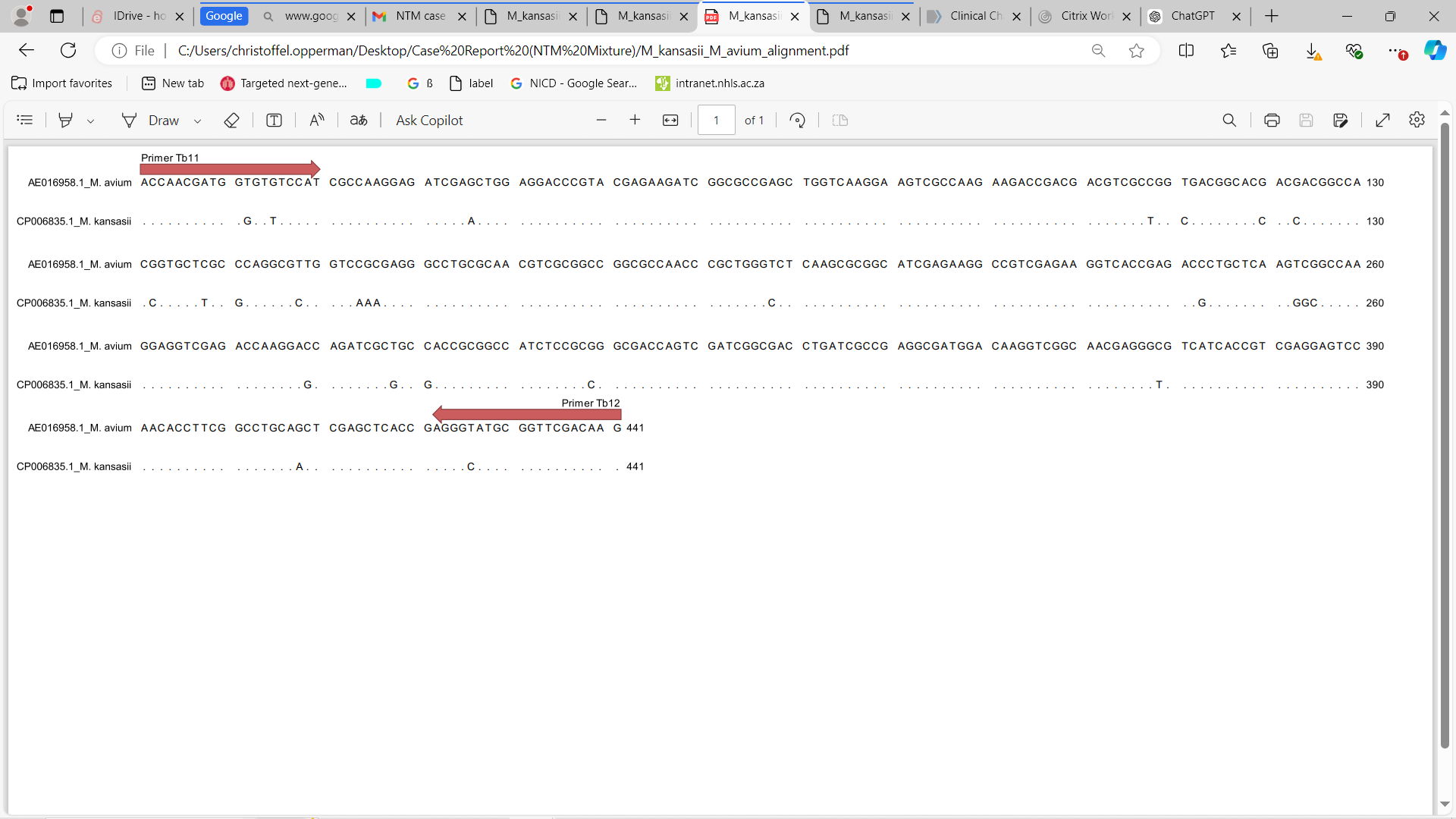


**Figure S2**. Differences in the genomic sequence of *Mycobacterium kansasii* and *Mycobacterium avium* at the binding sites of the Heat shock protein 65 (*hsp65*) forward and reverse primers, which may result in variations in primer binding affinity. Nucleotide differences between the two organisms are highlighted in the *M. kansasii* sequence. Primers: Tb11 Forward- 5’-ACCAACGATGGTGTGTCCAT-3’; Tb12 Reverse- 5’-CTTGTCGAACCGCATACCCT-3’
